# Fresh is best: Accurate SNP genotyping from koala scats

**DOI:** 10.1002/ece3.3765

**Published:** 2018-02-18

**Authors:** Anthony J. Schultz, Romane H. Cristescu, Bethan L. Littleford‐Colquhoun, Damian Jaccoud, Céline H. Frère

**Affiliations:** ^1^ GeneCology Research Centre University of the Sunshine Coast Maroochydore DC Qld Australia; ^2^ Global Change Ecology Research Centre University of the Sunshine Coast Maroochydore DC Qld Australia; ^3^ Diversity Arrays Technology University of Canberra Bruce ACT Australia

**Keywords:** diet, disease, koala, scat, SNP Genotyping

## Abstract

Maintaining genetic diversity is a crucial component in conserving threatened species. For the iconic Australian koala, there is little genetic information on wild populations that is not either skewed by biased sampling methods (e.g., sampling effort skewed toward urban areas) or of limited usefulness due to low numbers of microsatellites used. The ability to genotype DNA extracted from koala scats using next‐generation sequencing technology will not only help resolve location sample bias but also improve the accuracy and scope of genetic analyses (e.g., neutral vs. adaptive genetic diversity, inbreeding, and effective population size). Here, we present the successful SNP genotyping (1272 SNP loci) of koala DNA extracted from scat, using a proprietary DArTseq^™^ protocol. We compare genotype results from two‐day‐old scat DNA and 14‐day‐old scat DNA to a blood DNA template, to test accuracy of scat genotyping. We find that DNA from fresher scat results in fewer loci with missing information than DNA from older scat; however, 14‐day‐old scat can still provide useful genetic information, depending on the research question. We also find that a subset of 209 conserved loci can accurately identify individual koalas, even from older scat samples. In addition, we find that DNA sequences identified from scat samples through the DArTseq^™^ process can provide genetic identification of koala diet species, bacterial and viral pathogens, and parasitic organisms.

## 1 INTRODUCTION

Challenges to the conservation and management of rare, endangered, or cryptic species are compounded in part by the difficulty of gathering baseline population data (Boakes, Fuller, McGowan, & Mace, 2016). A lack of robust data on population size, distribution, and genetic diversity (Phillips, 2000; Sherwin, Timms, Wilcken, & Houlden, 2000) increases the uncertainty associated with management decisions, and the trade‐off between investing resources in data collection versus applied management is a complex issue (Grantham, Wilson, Moilanen, Rebelo, & Possingham, 2009; Jaramillo‐Legorreta et al., 2007; Knight et al., 2008; Whitten, Holmes, & MacKinnon, 2001). This is particularly true for rare and endangered species, which are generally characterized by small, reproductively isolated populations in fragmented landscapes (Channell & Lomolino, 2000; Drury, 1974; Gaston, 1994). Small, isolated populations are known to reduce individual fitness and heighten extinction risk (Lynch & Lande, 1993; Willi & Hoffmann, 2009; Willi, Van Buskirk, & Hoffmann, 2006), as increased inbreeding and genetic drift decrease standing genetic diversity (Keller & Waller, 2002; Spielman, Brook, & Frankham, 2004; Willi, Van Buskirk, Schmid, & Fischer, 2007).

As a result, the IUCN (International Union for Conservation of Nature) identifies genetic diversity as one of the key forms of biodiversity requiring conservation (McNeely, Miller, Reid, Mittermeier, & Werner, 1990). Traditional conservation planning for maintaining species biodiversity requires knowledge of habitat type, species assemblages, and ecological processes (Margules & Pressey, 2000; Pressey, Cabeza, Watts, Cowling, & Wilson, 2007). Similarly, management planning for the conservation of genetic diversity in wild populations requires accurate measurement of population genetic parameters. Specifically, conservation decision makers require reliable data on patterns of individual genetic diversity, dispersal, gene flow, population‐level diversity, levels of inbreeding, and effective population size (*N*_e_).

Patterns of connectivity and gene flow can be successfully investigated using both microsatellite markers (Hodel et al., 2016; Morin, Luikart, & Wayne, 2004) and SNP (single‐nucleotide polymorphism) markers (Van Inghelandt, Melchinger, Lebreton, & Stich, 2010). However, measuring inbreeding coefficients and effective population sizes is more suited to genome‐wide markers, for which SNPs are an increasingly popular choice (e.g., Bjelland, Weigel, Vukasinovic, & Nkrumah, 2013; Luikart, Ryman, Tallmon, Schwartz, & Allendorf, 2010; Saura et al., 2015). In addition, the higher resolution provided by SNP genotyping can also give less biased measures of genetic diversity than microsatellites (e.g., Munshi‐South & Kharchenko, 2010; Munshi‐South, Zolnik, & Harris, 2016). Generally, two to three SNPs are expected to provide the same power as one microsatellite, across a range of analyses (Fernández et al., 2013; Glover et al., 2010; Sellars et al., 2014). SNPs are also significantly more effective in species with low genetic diversity (Tokarska et al., 2009). It is, however, in the ability to genotype thousands of SNPs across the genome of a target species, that the power of SNPs over microsatellites lies (Davey et al., 2011).

Inbreeding and heterozygosity analysis comparisons have found that the addition of SNPs to microsatellite panels can increase accuracy, but adding microsatellites to SNP panels has little impact (Santure et al., 2010; Smouse, 2010). Effective population size estimation using SNPs has been successful across a range of species (The Bovine Hapmap Consortium, 2009; Corbin et al., 2010; McEachern, Eadie, & Van Vuren, 2007; Uimari & Tapio, 2011). For use in population and conservation genetic studies, SNPs can generally provide broader genome cover than microsatellites and mtDNA with equivalent statistical power (Morin et al., 2004).

Koalas (*Phascolarctos cinereus*) are cryptic, arboreal marsupials, with patchy distribution down the east coast of Australia, and listed as threatened in the northern parts of their range (Commonwealth 2012; DSEWPC 2013). Threats to koala populations include habitat loss and fragmentation, dog attacks, car strikes, wild fires, and *Chlamydia pecorum*‐related disease (Lunney, Gresser, Mahon, & Matthews, 2004; Lunney, Matthews, Moon, & Ferrier, 2000; Matthews, Lunney, Gresser, & Maitz, 2007; Melzer, Carrick, Menkhorst, Lunney, & John, 2000; Melzer, Cristescu, Ellis, FitzGibbon, & Manno, 2014; Polkinghorne, Hanger, & Timms, 2013). While there are no national‐scale studies of koala population statuses, studies in the northern parts of the koala's range suggest that habitat fragmentation has produced small, reproductively isolated populations which exhibit rapid genetic differentiation (Lee et al., 2010). Populations monitored in this region have declined by up to 80% over the last two decades, adding urgency to our understanding of koala genetic health (Rhodes, Beyer, Preece, & McAlpine, 2015).

Genetic studies of koala populations have traditionally relied on tissue or blood samples, either collected by capturing wild koalas (e.g., Fowler, Houlden, Hoeben, & Timms, 2000), or collecting samples from ill or injured animals bought into veterinary hospitals (e.g., Dudaniec et al., 2013). These studies have, for the most part, relied on microsatellites for genotyping and measuring diversity, using between 6 and 15 microsatellite loci (Cristescu et al., 2009; Dennison et al., 2017; Houlden, England, & Sherwin, 1996; Ruiz‐Rodriguez, Ishida, Greenwood, & Roca, 2014). This results in greatly reduced genetic comparability between studies, which for a low density, difficult to sample species, is a lost opportunity. Finding, capturing, and sampling wild koalas is costly in both time and money, while sampling sick or injured animals may bias sampling toward areas of increased human presence. However, the increasing use of noninvasive sampling has allowed for cheaper, easier genetic sampling across a range of species (e.g., okapi (*Okapia johnstoni*) (Stanton et al., 2016), wolves (*Canis lupus*) (Scandura, 2005; Stenglein, Waits, Ausband, Zager, & Mack, 2011), Spanish imperial eagles (*Aquila adalberti*) (Horváth, Martínez‐Cruz, Negro, Kalmár, & Godoy, 2005)), and more recently koalas (Wedrowicz, Karsa, Mosse, & Hogan, 2013). Scat sampling in particular, coupled with novel collection methods such as detection dog use, allows for widespread, unbiased sampling of koala genetic material. Microsatellite genotyping from koala scat is already available (Wedrowicz et al., 2013), albeit without a tissue DNA sample with which to compare the genotyping accuracy. While DNA isolated from tissue or blood is also likely to have low levels of error associated with them, a comparison of the error rates between blood/tissue DNA and scat DNA would prove a useful tool for assessing the practicality of using DNA from scat. A SNP panel has been developed for koalas (Kjeldsen et al., 2015), although thus far it has only been applied to tissue samples. SNP genotyping of noninvasively collected samples has been effective across a range of wild species including wolves (Kraus et al., 2015), river otters (*Lutra canadensis*) (Stetz et al., 2016), and European wildcats (*Felis silvestris silvestris*) (Nussberger, Wandeler, & Camenisch, 2014). With regard to koalas, while we know that DNA can be extracted from scats, we do not know whether this is sufficient to reliably genotype thousands of SNP markers, or at what scat ages this might be possible.

This is important as efficient, unbiased sampling of scat, and successful SNP analysis of the DNA contained therein would allow researchers to gather fine‐scale individual, population‐level, and landscape‐level data accurately and efficiently. Utilizing scat detection dogs, as previously mentioned, would be one way of accomplishing widespread scat sampling for such genetic analyses. This will provide enough high‐resolution genetic information to enable a comprehensive evaluation of koala genetic measures, specifically those mentioned above (*N*_e_, inbreeding, population diversity, and interpopulation diversity patterns). With a greater depth of genetic information, we will be far more able to plan conservation programmes and interventions to best maintain koala genetic diversity, which until now has been very difficult to assess.

Here, we used DArTseq^™^ to test the feasibility of SNP genotyping using DNA extracted from koala scats. In order to assess the effect of scat age on genotyping results, we extracted DNA from scats of different ages. In particular, and in addition to previous studies, we compared results from fecal DNA samples to DNA from blood to test fecal genotyping accuracy. DArTseq^™^ technology was chosen for this analysis in part due to its high repeatability and standardization of SNP loci used for genotyping. Multiple samples across multiple analyses can be genotyped using the same complexity reduction method, allowing for maximum comparability across studies and individuals.

## 2 MATERIALS AND METHODS

Fresh scats (<6 hr old) were collected from five captive koalas (three females and two males) by watching koalas as they defecated and retrieving the pellets from the ground. Sampled koalas were resident at Wildlife HQ Zoo in Woombye, Queensland. Whole blood samples (2 ml) were taken from each animal during regular veterinarian examinations. Based on zoo records, these five animals are all from different areas in Queensland, and two individuals are related (a father and daughter).

To establish the effectiveness of SNP genotyping from scat extractions, two scats per individual were stored on toothpicks stuck in a Styrofoam board in the laboratory, under ambient light and temperature (approximately 28°C). Scats were aged under these conditions over the course of two weeks. Scats were harvested for DNA isolation on day two and day 14 postcollection. At both sampling points, DNA was isolated from two scats per individual. Due to koalas sharing enclosure space, there was a misidentification of a scat which was only discovered through genotyping results. One of the two‐day‐old scats from Koala 4 was actually from Koala 2 and was renamed as such.

### 2.1 DNA isolation—blood

DNA was isolated from each koala blood sample using the Wizard^®^ Genomic DNA Purification Kit (Promega) following the manufacturer's “Isolating Genomic DNA from Whole Blood (300 μl sample volume)” protocol. Isolates were stored at −80°C.

### 2.2 DNA isolation—scat

Koala DNA was isolated from intestinal epithelial cells on sampled scats. Epithelial cells from the surface of each scat were collected by slicing off the outer‐most layer of the scat using a scalpel. These surface slices were then used to extract koala DNA using the QIAamp DNA Stool Mini Kit (Qiagen), following an adapted version of the manufacturer's protocol “Isolation of DNA from Stool for Human DNA Analysis,” as follows: At cell lysis stage, 1.8 ml Buffer ASL was added, vortexed for one minute, and centrifuged at full speed for two minutes. For each isolate, 2 μl (100 ng/ml) RNase A (Qiagen) was added and incubated at 37° C for 30 min. The quantity of total DNA in each scat isolate was measured using a Thermo‐Scientific Nanodrop 2000 Spectrometer. DNA isolates were stored at −80° C.

Koala dietary species are known to contain volatile compounds and phenolics, which are subsequently excreted in scats (Eberhard, Mcnamara, Pearse, & Southwell, 1975). Some of these compounds (including 1,8‐cineole and terpinene‐4‐ol) have been shown to contribute to cell membrane damage (Carson, Hammer, & Riley, 2006). Additionally, phenolics are known to contribute to accelerated DNA degradation (Khan & Hadi, 1998) and may also inhibit PCR processes (Kreader, 1996). QIAamp DNA Stool Mini Kit (Qiagen) specifically includes InhibitEx tablets specifically designed to remove such PCR inhibitors during DNA extraction.

### 2.3 SNP genotyping

Two DNA isolates for each individual, for each sampling point, were used for SNP genotyping. SNP genotyping was conducted by Diversity Arrays Technology, Canberra, using proprietary DArTseq^™^ technology. DArTseq^™^ technology has been tested and used successfully for a wide range of genomic studies across a variety of vertebrate species (Melville et al., 2017). Examples of this include Cunningham's skinks (*Egernia cunninghami*) (Ofori, Beaumont, & Stow, 2017), North American green frog (*Rana clamitans*) (Lambert, Skelly, & Ezaz, 2016), trout cod (*Maccullochella macquariensis*) and Murray cod *(Maccullochella peelii*) (Couch, Unmack, Dyer, & Lintermans, 2016), yellowfin tuna (*Thunnus albacares*) (Grewe et al., 2015), eastern yellow robin (*Eopsaltria australis*) (Morales et al., 2017), and southern fiddler rays (*Trygonrrhina dumerilii*) (Donnellan et al., 2015).

DArTseq^™^ represents a combination of DArT complexity reduction methods and next‐generation sequencing platforms (Courtois et al. 2013; Cruz, Kilian, & Dierig, 2013; Kilian et al., 2012; Raman et al., 2014). Similar to DArT methods based on array hybridizations, the technology is optimized for the specific organism and application by selecting the most appropriate complexity reduction method. In this study, the combination of PstI and SphI restriction enzymes (RE) performed better in polymorphism detection efficiency. When genome complexity reduction methods are compared, those showing increased percentages of repetitive elements, skewed size ranges, or nonideal numbers of fragments are avoided.

DNA samples were processed in digestion/ligation reactions (Kilian et al., 2012), ligating two adaptors corresponding to the combination of RE overhangs. The PstI‐compatible adapter includes the barcode. The barcodes are of different length varying between 4 and 8 bp, this was designed to stagger the sequencing start position, similar to the method reported by Elshire et al. (2011). The reverse adapter contained the SphI‐compatible overhang sequence.

The PstI‐SphI fragments were amplified by adapter‐mediated PCR as follows: initial denaturation of 94° C for 1 min, followed by 30 cycles of denaturation (94° C for 20 s), annealing (58° C for 30 s), and extension (72° C for 45 s), with final extension phase of 72° C for 7 min. The PCR primers were designed to add the required sequences for enabling sequencing in a single‐read Illumina flowcell. Equimolar amounts of amplification products from each sample were bulked and applied to c‐Bot (Illumina) bridge PCR followed by 77 cycles of single‐read sequencing on Illumina Hiseq2500 (Illumina).

The resulting sequences generated were processed using proprietary DArT analytical pipelines. The primary pipeline filtered out poor quality sequences, while applying more stringent selection criteria to the barcode region. In this way, assignment of sequences to specific samples was very reliable. Identical sequences were then collapsed into “fastqcol” files for use in secondary pipeline analysis, using DArT PL's proprietary SNP and SilicoDArT (presence/absence of restriction fragments in representation) calling algorithms (DArTsoft14).

For SNP calling, all tags from all libraries included in the DArTsoft14 analysis are clustered using DArT PL's C++ algorithm at the threshold distance of 3, followed by parsing of the clusters into separate SNP loci using a range of technical parameters, especially the balance of read counts for the allelic pairs. Additional selection criteria were added to the algorithm based on analysis of approximately 1,000 controlled cross populations. Testing for Mendelian distribution of alleles in these populations facilitated selection of technical parameters discriminating well true allelic variants from paralogous sequences. In addition, multiple samples were processed from DNA to allelic calls as technical replicates, and scoring consistency was used as the main selection criteria for high quality/low error rate markers. Calling quality was assured by high average read depth per locus. This process is similar to that used in published literature using DArTseq^™^ SNPs from animal genetic samples (e.g., Couch et al., 2016; Donnellan et al., 2015).

Sequences identified during the DArTseq^™^ process were run through the National Center for Biotechnology Information's (NCBI) BLAST (basic local alignment search tool) (Altschul, Gish, Miller, Myers, & Lipman, 1990) to investigate possible dietary or disease‐related DNA that was included in scats.

### 2.4 Assessing error, descriptive analysis, and potential sexing loci

For each genotyped sample, percentage missing data were calculated, and genotype comparison between blood DNA results and scat DNA results for both scat ages was used to assess allelic dropout and false alleles. SNP loci overlap between blood DNA genotypes and scat genotypes were calculated, as well as loci overlap across all blood samples (population loci overlap). In this context, overlap refers to the percentage of SNP loci with successful genotype reads across all specified samples. Thus, high overlap between samples suggests that a high percentage of the 1272 SNP loci produced genotype reads across all the specified samples being compared. As the sex of all koala individuals was known for this study, putative sex‐linked SNP loci were also identified.

Sequencing depths for both reference and SNP alleles, for each locus, for each sample were investigated, and average sequencing depth for blood DNA, two‐day‐old, and 14‐day‐old scat samples were calculated.

### 2.5 Genetic analyses and visualization

Analyses of allelic frequency and genetic distance between all samples were conducted in GenAlEx 6.503 (Peakall & Smouse, 2006, 2012). To assess whether individuals could be accurately identified using genotypes from scat DNA extractions, neighbor‐joining trees were constructed for a variety of loci subsets, based on error rates (i.e., missing data, scat genotype different to blood genotype), sequencing depth, overlap between two‐day‐old and 14‐day‐old samples, and excluding homozygous SNP loci. This enabled us to identify a suite of accurate SNP loci appropriate for successful individual identification from scat samples. Neighbor‐joining trees were constructed using FAMD (Fingerprint Analysis with Missing Data) software (Schlüter & Harris, 2006) and visualized in MEGA 7 (Kumar, Nei, Dudley, & Tamura, 2008; Kumar, Stecher, & Tamura, 2016).

To further test the utility of the 209 SNP panel selected for individual identification, we calculated the probability of identity for unrelated individuals (*P*_ID_) and the more conservative probability of identity for full siblings (*P*_IDsibs_). The probability of identity measures the probability that two individuals drawn randomly from the population will have identical genotypes across a given marker panel (Lorenzini, Posillico, Lovari, & Petrella, 2004; Mills, Citta, Lair, Schwartz, & Tallmon, 2000; Waits, Luikart, & Taberlet, 2001). We used GenAlex 6.503 (Peakall & Smouse, 2006, 2012) and included all samples used in neighbor‐joining tree analysis (*n *= 19).

## 3 RESULTS

While 100% of the two‐day‐old scat samples were successful, only 70% of 14‐day‐old samples provided high enough quality DNA for successful library construction. The low‐quality samples were therefore excluded from subsequent analyses. DNA concentrations from scat samples in this study were found to be comparable to a similar study in which koala fecal DNA was isolated for microsatellite genotyping (Wedrowicz et al., 2013), suggesting that the DNA isolation methods utilized in this study provide comparable results to extraction methods used in other studies. The average DNA concentration for two‐day‐old scat in this study was similar to that found in the comparison study (two‐day‐old‐scat average: 10.94 ng/μl; <30‐hr‐old scat in comparison study: 11 ng/μl), while the average for 14‐day‐old scat in this study was found to be higher than that of 28‐day‐old scat in the comparison study (14‐day‐old scat average: 3.2 ng/μl; 28‐day‐old scat in comparison study: 2.2 ng/μl). DNA concentrations for each two‐day‐old and 14‐day‐old scat samples are reported in Table 1. The estimated size ranges for the amplified fragments were between 20 bp and 700 bp, with a peak between 120 bp and 200 bp. Additionally, having access to the koala genome (when published) will allow for better mapping and calculation of fragment lengths.

**Table 1:** SNP loci overlap between scat DNA and blood DNA samples, percentage of missing genotype data for all samples, percentage of null alleles read in scat samples in comparison with blood DNA template, percentage of incorrect genotype reads in scat samples in comparison with blood DNA reads, and total DNA concentrations from scat extraction reactions. Null allele percentages and incorrect genotype read percentages are a percentage of total loci with no missing data. DNA concentration readings include all DNA extracted during reaction and so will also include nontarget DNA

Sample	Sample type	Loci overlap with blood DNA sample (%)	Missing data (%)	Null alleles in scat samples (%)	Incorrect genotype reads in scat samples (%)	Total DNA concentration (ng/μl)
Koala 1	Blood		0.24			
Koala 2	Blood		0.31			
Koala 3	Blood		0.16			
Koala 4	Blood		0.08			
Koala 5	Blood		1.89			
Koala 1 Day2a	Scat	94.6	8.81	10.17	5.95	5.65
Koala 1 Day2b	Scat	93.6	24.92	11.31	7.75	30.3
Koala 1 Day14a	Scat	72.3	27.75	21.98	1.63	9.6
Koala 1 Day14b	Scat	34.5	65.64	27	2.29	7.25
Koala 2 Day2a	Scat	99.5	49.21	23.53	1.39	16.3
Koala 2 Day2b	Scat	13.3	8.88	17.26	0.78	6.85
Koala 2 Day2c	Scat	99.5	0.47	18.88	26.22	8.35
Koala 3 Day2a	Scat	94.3	5.66	10.67	1.17	5.7
Koala 3 Day2b	Scat	75.2	24.84	19.77	2.62	6.9
Koala 3 Day14a	Scat	50.8	0.47	4.42	1.58	0.55
Koala 3 Day14b	Scat	91.1	86.71	26.04	14.2	0.25
Koala 4 Day2a	Scat	99.8	0.16	2.91	1.65	5.6
Koala 4 Day14a	Scat	97.5	2.52	9.11	1.69	2.25
Koala 4 Day14b	Scat	99.8	0.24	2.99	1.81	1.93
Koala 5 Day2a	Scat	95.4	4.64	7.75	2.97	13.4
Koala 5 Day2b	Scat	97.3	2.67	5.49	4.04	10.35
Koala 5 Day14a	Scat	15.6	84.43	19.7	11.11	0.6

### 3.1 SNP loci from blood DNA

DArTseq^™^ technology identified 1272 SNP loci. As koalas are a nonmodel species, reference alleles and SNP alleles for each locus were assigned arbitrarily—in most cases, reference alleles were indicated as the allele that was most frequent across all samples for that locus. Of the 1272 loci identified, 1247 loci (98.0%) were found to overlap across blood DNA samples from all five individuals. One hundred and sixty‐nine loci (13.6%) were found to be homozygous across all individuals and so are uninformative for this sample size.

### 3.2 Potential sexing loci

Of the 1272 loci identified, 26 potentially sex‐linked candidate loci were found. That is, loci which were present across all individuals, and varied consistently in genotype between males and females. For example, locus ID #12495936 (Appendix S2: Table S1) showed alleles TG for both male individuals and alleles TT for all female individuals during genotype calling.

### 3.3 Blood genotyping to scat genotyping analysis

In comparison with DNA extracted from blood, two‐day‐old scat DNA had higher, and more consistent SNP loci overlap (maximum overlap: 99.8%; minimum overlap: 13.3%; median overlap: 95.0%) than 14‐day‐old scat DNA samples (maximum overlap: 99.8%; minimum overlap: 15.6%, median overlap: 72.3%) (Table 1). High overlap percentage means more loci were successfully genotyped in both blood DNA and scat DNA, suggesting that fresher scats provided an average genotyping picture closer to that of blood DNA than older scats.

**Figure 1:**
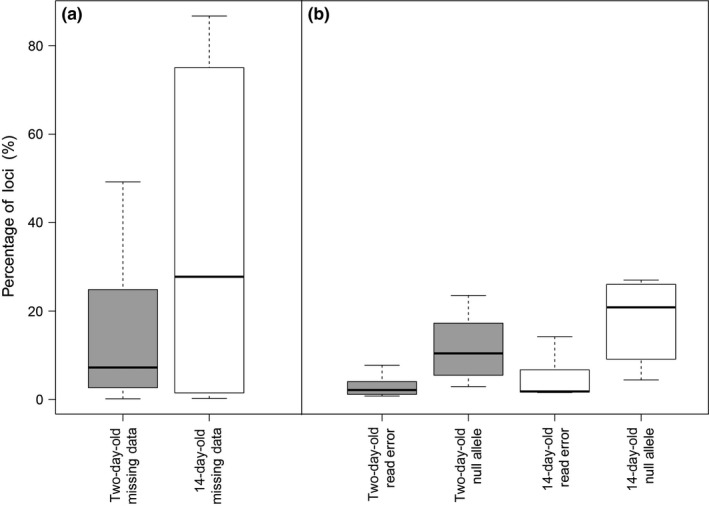
Two‐day‐old versus 14‐day‐old scat DNA (a) missing data and (b) genotyping error, when compared to template genotype from blood. Missing data (a) shows percentage of loci (*n *= 1272) which do not provide any read data in scat samples. Read error and null allele (b) show percentage of remaining loci which do not match blood template genotype due to incorrect read or allelic dropout. Sample size: two‐day‐old samples: *n *= 10; 14‐day‐old samples: *n *= 7

When comparing error rates and types between fresher and older scats, two‐day‐old scat DNA samples had on average less missing data, and less variability in missing data, than 14‐day‐old scat DNA samples (Figure 1, Table 1). Interestingly, among loci that do provide data, the difference in read error rates or null allele rate between two‐day‐old and 14‐day‐old scat samples was low, suggesting that differences in genotyping results between scat ages were driven by missing data over other error types.

### 3.4 Average read depths

**Figure 2:**
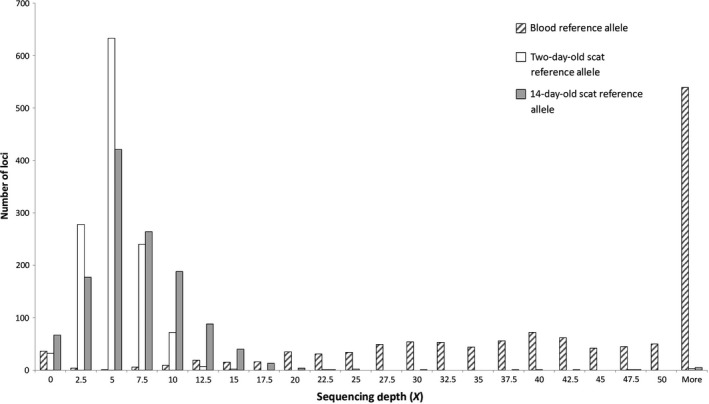
Distribution of reference allele sequencing depths of 1272 SNP loci for blood DNA extractions, two‐day‐old scat DNA extractions, and 14‐day‐old scat DNA extractions. Average sequence depth across all loci: Blood: Ref allele—49*X*, SNP allele—31*X*; Two‐day‐old scat: Ref allele—4.3*X*, SNP allele—3.2*X*; 14‐day‐old scat: Ref allele—6.1*X*, SNP allele—3.8*X*

Read depth refers to the number of times a SNP locus has been sequenced and mapped during the genotyping process (Fumagalli, 2013). Genotypes are then called from these reads, where sites with higher numbers of reads are likely to have higher accuracy in genotype calling. Conversely, loci with lower numbers of reads are likely to exhibit non‐negligible errors in genotype calling (Crawford & Lazzaro, 2012). Read depth is then an important measure of SNP quality when assessing the likely accuracy of genotype calling. The average read depth for all 1272 loci differed greatly between blood DNA samples and scat DNA samples (Figure 2), with blood samples having on average nine times greater read depth per locus. Fourteen‐day‐old samples showed on average slightly higher (Reference allele—6.1*X*; SNP allele—3.8*X*) read depths than two‐day‐old (Reference allele—4.3*X*; SNP allele—3.2*X*) samples. However, for all (*n *= 7) 14‐day‐old samples, there were 206 loci present which did not provide genotype reads in any samples. In comparison, for two‐day‐old scat samples, there were only nine loci which contained missing information across all samples.

### 3.5 Allele frequency

For 1272 SNP loci across five individuals, 559 loci (44%) had a minor allele frequency of either 0% or 10%. This may be an artifact of our small sample size (Appendix S1: Figure S1).

### 3.6 Genetic distance

To identify a panel of SNP loci useful for identifying individual koalas from scat‐extracted DNA, loci were selected based on high sequencing depth, low error rates (i.e., missing data, null alleles, and false allele reads), loci overlap between two‐day‐old and 14‐day‐old scat samples, and homozygous loci. For two‐day‐old scat samples (*n *= 10) and 14‐day‐old scat samples (*n *= 7), SNP loci were excluded if genotypes were homozygous across samples, sequencing depth for reference allele was <5*X*, missing data were found in more than three samples, and if scat genotype did not match blood genotype in more than three samples. These subsets of loci were then compared between scat ages, and only those loci common to both scat age subsets were included in the neighbor‐joining tree. Furthermore, scat DNA samples missing more than 50% data across the selected 209 loci were also excluded, regardless of age.

**Figure 3:**
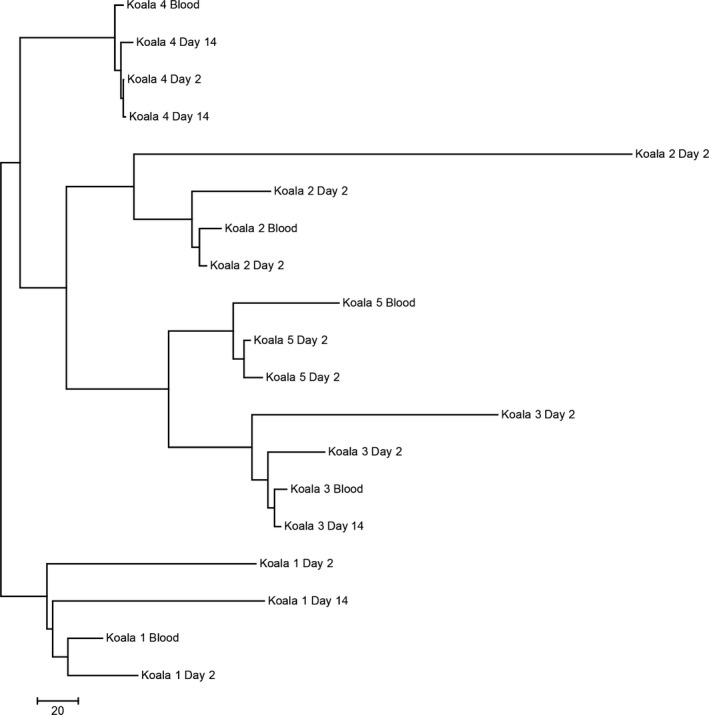
Neighbor‐joining tree of genetic distances using 209 highly conserved SNP loci for blood and scat DNA samples. Loci selected for genetic distance calculation was based on sorting for sequencing depth, error rates, and homozygous loci. Scat DNA samples with missing information at more than 50% of loci were excluded from this analysis

The resulting neighbor‐joining tree (Figure 3) showed greater genetic difference between individuals than within individuals. This suggests that the 209 loci panel identified could be used to differentiate between individual koalas at a genetic level, even when scats are 14 days old prior to sampling. Interestingly, Koala 5 is the daughter of Koala 3, which cluster together on the joining tree, suggesting that first‐degree relatedness between individuals may also be identifiable from using SNP markers on DNA from scat. The specific loci included in this panel are identified in the DRYAD online data repository submission.

Average *P*_ID_ and *P*_IDsibs_ measures were calculated for the 19 samples used in the final neighbor‐joining tree, using the 209 loci panel selected for individual identification. Average *P*_ID_ was 3.5 × 10^−52^, while the more conservative *P*_IDsibs_ was 1.3 × 10^−26^. These values are considered low for probability of identity calculations (<0.0001) and suggest a very low probability of two individuals with identical multilocus genotypes being drawn randomly from the population. Conversely, then, individuals with identical genotypes found in the population would be assumed to be resampling of the same individual. Using the subsetted SNP marker panel of 209 loci for *P*_ID_, we require only ten loci to reach a 1 in 100 chance of randomly drawing two individuals with the same genotype by chance, and 20 loci to reach a 1 in 10,000 chance of drawing the same. For the more conservative *P*_IDsibs_ measure, we require twenty loci and thirty‐nine loci, respectively. Hence, we expect our 209 loci marker set to have adequate discriminatory power in accurately identifying individuals.

### 3.7 BLAST results

Running DNA sequences identified during the DArTseq^™^ process through BLAST revealed dietary and disease information (see Appendix S2: Table S2). In relation to diet, we identified multiple BLAST hits for *Eucalyptus grandis* (41 predictive BLAST hits) in scats (a common food tree known to be provided by zoo staff for koalas, J. Schenk, Wildlife HQ CEO, pers. comm. 2017). This is a known koala food tree (Lunney et al., 2000) and suggests that individual‐specific dietary information may be accessible through genetic analysis of scats. From a disease perspective, BLAST results turned up multiple complete sequences of koala retrovirus (KoRV) isolates (four BLAST hits). Evidence of KoRV was most noticeable in blood samples, although there were also positive hits in scat samples. In addition, there were BLAST hits for the parasitic nematode *Parastrongyloides trichosuri* (four BLAST hits), whose natural hosts are possums of the *Trichosurus* genus (Grant et al., 2006). We also found evidence of *Pseudomonas aeruginosa* bacteria (13 BLAST hits). This is a known pathogen which has been associated with pneumonia in wild koalas (McKenzie, 1981). These results indicate that the process of genotyping koalas from scat DNA may also allow for much greater information on diet and disease (bacterial, viral, and parasitic) presence than previously thought.

## 4 DISCUSSION

DNA extracted from a single koala scat can provide enough high‐quality DNA to successfully genotype individuals using 1272 SNP markers, without the multitube approach required in many noninvasive studies (Regnaut, Lucas, & Fumagalli, 2006). Additionally, this is the first time that koala fecal DNA has been compared to blood DNA to test genotyping accuracy. We demonstrate that powerful next‐generation population genetics approaches are possible for koala fecal DNA, allowing for a greater variety of genetic analyses based on noninvasive samples taken from wild koalas.

While genotyping errors, mostly due to missing data at underperforming loci, varied greatly between two‐day‐old and 14‐day‐old scat, average sequencing depth did not. Sequencing depth from fecal DNA was greatly reduced when compared to that of blood DNA samples, but average depth across all scat samples was still 4.6*X* (reference allele average: 5.6*X*; SNP allele average: 3.6*X*). Next‐generation sequencing data simulation by Fumagalli (2013) suggest that highly precise detection of polymorphic sites can be achieved by genotyping small sample sizes at high sequencing depth (*n *= 20, depth = 50*X*, precisio*n *= 1). However, genotyping larger sample sizes at lower sequencing depths can provide comparable results (>75% precision). For example, a sample size of 500 individuals sequenced at 2*X* depth can maintain precision of 0.778 ± 0.0641, similar to a sample size of 100 individuals sequenced at 10*X* depth (precisio*n *= 0.779 ± 0.0441).

Sampling larger sample sizes at lower depth may be particularly well suited to scat DNA analysis. For example, detection dog scat sampling allows to greatly increase our sample size across the target landscape (Cristescu et al., 2015), wherein the lower average sequencing depths we see in fecal DNA analyses can still provide precise polymorphic reads. For analyses investigating population‐level genetic trends (e.g., population structure, interpopulation genetic diversity, and gene flow), we can therefore utilize all 1272 loci identified here, as larger sample sizes will balance out lower sequencing depths.

For analyses which require accurate individual identification, we can then focus on the smaller sample sizes and higher sequencing depths recommended by Fumagalli (2013).

Here, we have excluded SNP loci with low sequencing depths and high error rates, to identify a suite of loci that perform well on scats up to 14 days old, allowing for accurate individual‐level analysis for samples that may have partially deteriorated. This panel of 209 SNP loci can be used in individual‐based genetic analyses, such as determining inbreeding coefficients and effective population sizes, which are of particular importance to the conservation of genetic diversity. Additionally, the use of SNP genotyping in repeatedly identifying individual animals opens the door for mark–recapture studies to estimate koala population sizes—one of the most difficult ecological metrics to assess in koalas, and one of the most crucial for making informed conservation decisions (Lurz, 2008; Phillips, 2000; Shaffer, 1981). Using this panel, we are able to confirm the first‐degree relatedness of two koalas in this study, identifying Koala 3 as the father of Koala 5. Additionally, by removing samples with high levels of missing data (higher than 50% missing data, invariably 14‐day‐old samples), we can ensure that the individual identification results are accurate across all individuals. By utilizing blood DNA as a template in this study, we could assess how age may influence the effectiveness of genotyping and also established a threshold for excluding samples from analyses that require individual‐level accuracy. Furthermore, the utility of this 209 loci marker panel was reinforced by the *P*_ID_ and *P*_IDsibs_ results. The very low probability (<0.0001) of incorrectly identifying two independent individuals as the same individual using this marker panel attests to its strong discriminatory power. Given that only thirty‐nine loci were needed to achieve satisfactory discrimination between individual samples (i.e., <0.0001) for the *P*_IDsibs_ measure, we feel confident that this panel can reliably identify individuals in the typically larger sample sizes used in analyses of wild populations.

With regard to other information captured during the genotyping process, the presence of dietary information (*E. grandis*) provides evidence that individual koala diet could be assessed alongside genotyping. As koalas are known to spend time in nonfood trees (Briscoe et al., 2014), simple presence in a tree is not always indicative of diet, and researchers currently have to rely on time‐consuming leaf cuticle analyses (Melzer et al., 2014). A tailored approach to identifying the food tree preferences of individual koalas across a landscape could provide large‐scale ecological information currently unavailable to researchers. Testing of the sensitivity of genetic approaches to changes in diet may be the next step in this research, but these results are the first evidence we know of, of koala dietary indicators being genetically identified in scat. Furthermore, the addition of information on disease presence for bacteria, viruses, and parasitic invertebrates adds yet another layer of information on koala health that is currently difficult and costly to assess. Obviously, BLAST searches will only register sequences already in the NCBI databases, and so the BLAST hits for *Parastrongyloides trichosuri*, the parasitic possum nematode, are possibly identifying a koala‐specific nematode from the same genus, which has not yet been described. It is interesting to note that there is no evidence of *C. pecorum* in any extracted DNA, but given that the five individuals assessed in this study are animals bred in captivity, it should not be surprising that they are *C. pecorum*‐free. That multiple BLAST hits for each of these organisms were detected adds strength to our proposal that these are accurate identifications, supported by biological rationale for their presence. Further study into the relationship between the presence of such pathogens in blood and scat and the health of the individual koala is obviously required. However, the fact that such wide‐ranging bacterial, viral, and parasitic organisms can be detected through the DArTseq^™^ process is encouraging for assessing the health of wild koalas.

While there is no doubt that fresh is best when it comes to noninvasive scat sampling for genetic analyses, the limitations of collecting scat from wild populations, even with the advances in speed and accuracy introduced by detection dogs, means that it may not always be possible to sample scats within the first two days. Our research, however, shows that older scats can still be useful, depending on the research question and project design. It is also important to remember that while some 14‐day‐old scats provided enough high‐quality DNA for individual identification in this study, scats were aged under laboratory conditions, and so an upper limit of 14 days may not be realistic for scats collected from wild koalas. Ultraviolet light, rain, ground cover vegetation, and phenolics and volatile organic compounds released from koala scats as they decompose may all lead to rapid koala fecal DNA degradation. Indeed, this may result in faster DNA degradation in koala scats than is often found in other noninvasively sampled species, and so under ideal circumstances, the freshest scat should be sought wherever possible (Cristescu, Goethals, Banks, Carrick, & Frère, 2012; Wedrowicz et al., 2013). While very fresh koala scat is obviously ideal for genotyping, there is most likely a good compromise in practicality of sampling and quality of results somewhere between two‐day‐old scat and 14‐day‐old scat. Fortunately, koala scat age can be estimated by sight with a degree of accuracy, with pellets <14 days old recognizable by their shiny, uncracked patina, and strong eucalypt smell (Sullivan, Norris, & Baxter, 2002).

Across different scat ages, it is also important to consider the two possible causes of poor genotyping results. Firstly, that insufficient high‐quality DNA is extracted from scat samples to allow for library construction. In these cases, as seen with 30% of 14‐day ‐old scat samples in this study, no information can be produced from such samples. When this occurs, optimization of the DNA extraction process, and inclusion of PCR facilitators such as BSA (bovine serum albumin), may yield improved results. Other alternatives might include extracting DNA from replicate scats for older samples, to ensure higher DNA yield. Despite this, our study shows that 70% of 14 day old scats contained sufficient DNA to construct libraries for DArTseq^™^ SNP genotyping, thus validating the DArTseq^™^ technology for use in this application.

The second problem may arise whereby extracted DNA is already degraded (due to environmental factors, scat contents, volatile compounds etc.). This can result in the presence of missing data (null alleles and allelic dropout), as evidenced in the successfully amplified 14‐day‐old scat samples in this study. That this missing genotype data is due to DNA degradation rather than inefficiencies in the extraction process is further supported by the favorable comparison of DNA concentrations between the 14‐day‐old samples in this study and older samples in a similar study (Wedrowicz et al., 2013). Furthermore, these DNA concentration results point to the utility of the DNA extraction process used in this study, suggesting most errors are due to degraded DNA. As this DNA degradation will most likely have occurred prior to extraction, optimization of the amplification and genotyping processes may be necessary to achieve optimal results. Possible options here include targeting smaller fragments from amplification. Further study into which factors are most likely to introduce error into genotyping results, and how to specifically target them, would also allow for better future project design.

With regard to collecting scat from wild populations, detection dogs are increasingly used in koala conservation (Cristescu et al., 2015). While dogs trained to find scat of all ages are useful for identifying koala habitat, scats older than two weeks are not as suitable for genetic analysis using the methods outlined in this study. Thus, dogs trained specifically to find fresher scat may be a useful addition to conservation research and could greatly increase the number of genetic samples collected from wild koala populations. To this end, the authors are currently training a detection dog to prioritize finding fresh (<1‐week‐old) koala scat. This, coupled with growing citizen science programs whereby members of the public collect and freeze fresh scat for researchers, can provide high‐quality DNA samples for SNP genotyping and subsequent analysis. These novel sources of genetic samples can allow for large enough sample sizes to study important aspects of wild koala population genetics, which have been previously unavailable to researchers. Additionally, the potential to gather not only koala genetic information, but also dietary and disease information using this same process makes the use of koala scat for next‐generation genetic analyses an increasingly powerful tool.

When it comes to testing novel applications of genotyping methods, the question of sample size is always an important consideration. While more is invariably better, in this case, the sample size of five individuals is sufficient as a proof of concept for the application of this methodology. There are a number of reasons for this: Firstly, the DArTseq^™^ protocol utilized in this study is a well‐documented methodology. It has been used effectively across a range of species and specifically recommended for vertebrate studies (Melville et al., 2017). In particular, the standardization of loci genotyped across samples, and the repeatable complexity reduction methods provide a reliable and widely applicable methodology. This holds true regardless of samples size. Secondly, the highly conserved 209 loci used for individual identification perform well for both individual identification analyses conducted. In neighbor‐joining tree analysis, all samples from the same individual group together in neighbor‐joining tree analyses. Furthermore, there was accurate discrimination between scat DNA samples from the father–daughter pairing. The probability of identity analyses runs in this study also support these results. Thus, we are confident of the power of the 209 SNP loci panel to determine identity in larger populations.

As with applying published methodologies to any new context, it is always valuable to consider the possible limitations of the application and the conditions under which it has been tested. Regardless of this, this study provides sufficient evidence that high‐quality koala DNA can be extracted from scats to facilitate SNP genotyping using the DArTseq^™^ methodology. The increased power provided by SNP genotyping for genetic analysis ensures that important aspects of koala population ecology and genetics can be adequately assessed before conservation decisions are made, allowing for more accurate interventions and management strategies.

## DATA ACCESSIBILITY

Blood DNA and scat DNA genotypes for koalas used in this study have been uploaded to Dryad repository. https://doi.org/10.5061/dryad.mq0gb.

## CONFLICT OF INTEREST

None declared.

## AUTHOR CONTRIBUTIONS

AS, CF, and RC designed this research project. AS and BL‐C performed laboratory work. AS, DJ, and CF performed data analysis. AS, CF, RC, and DJ wrote the manuscript.
