# Supplementary figures and images for: Fresh is best: Accurate SNP genotyping from koala scats
Source: Ecol Evol. 2018 Feb 18;8(6):3139–51. doi: 10.1002/ece3.3765 (PMC5869377; doi:10.1002/ece3.3765)

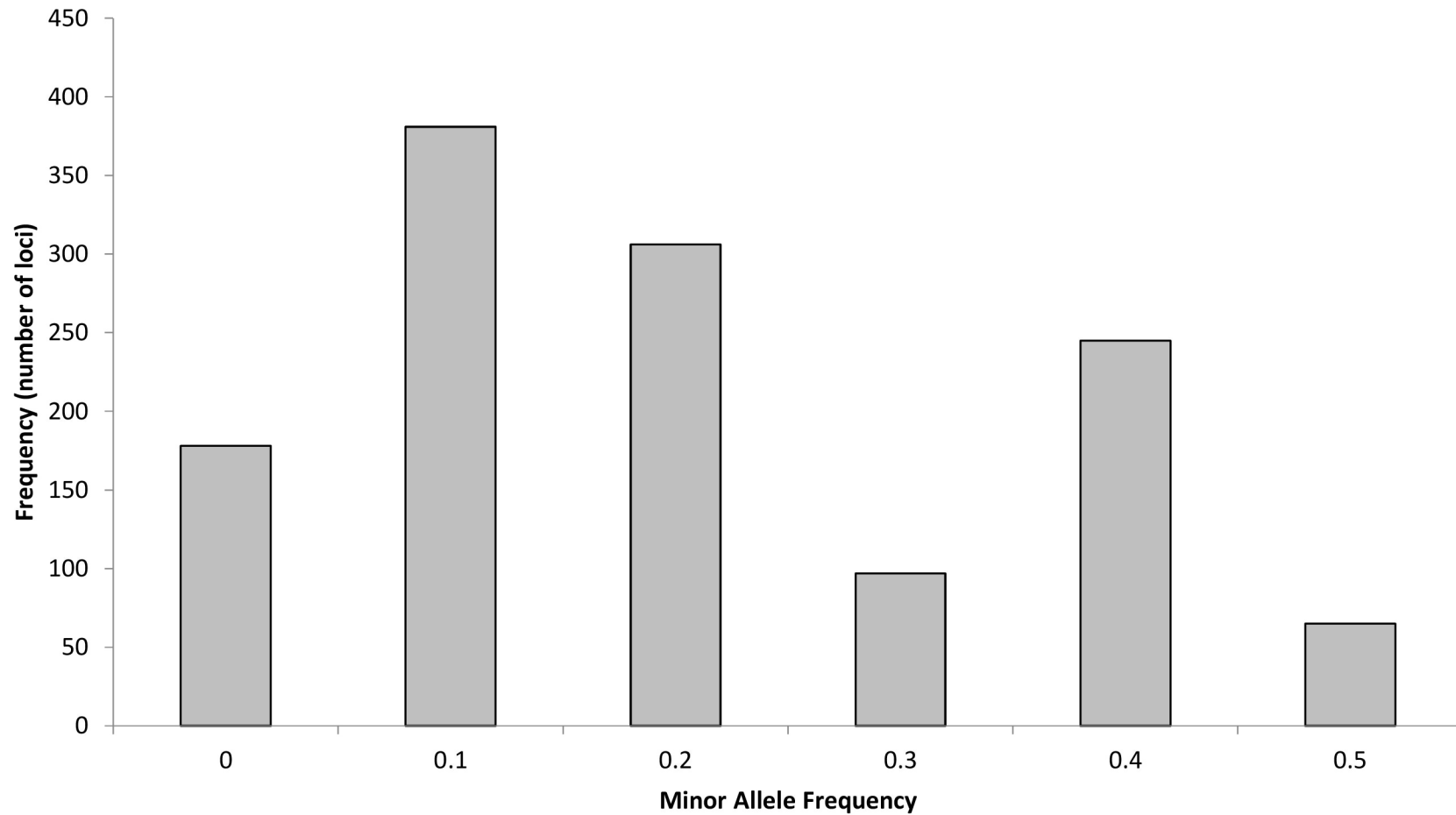

Supplement: Supplementary file 1 [file ECE3-8-3139-s001.pdf]
