# Supplementary material for: Fresh is best: Accurate SNP genotyping from koala scats
Source: Ecol Evol. 2018 Feb 18;8(6):3139–51. doi: 10.1002/ece3.3765 (PMC5869377; doi:10.1002/ece3.3765)
Supplement: Supplementary file 2 [file ECE3-8-3139-s002.docx]

APPENDIX

**Appendix Table 1.** Potentially sex-linked koala SNP loci
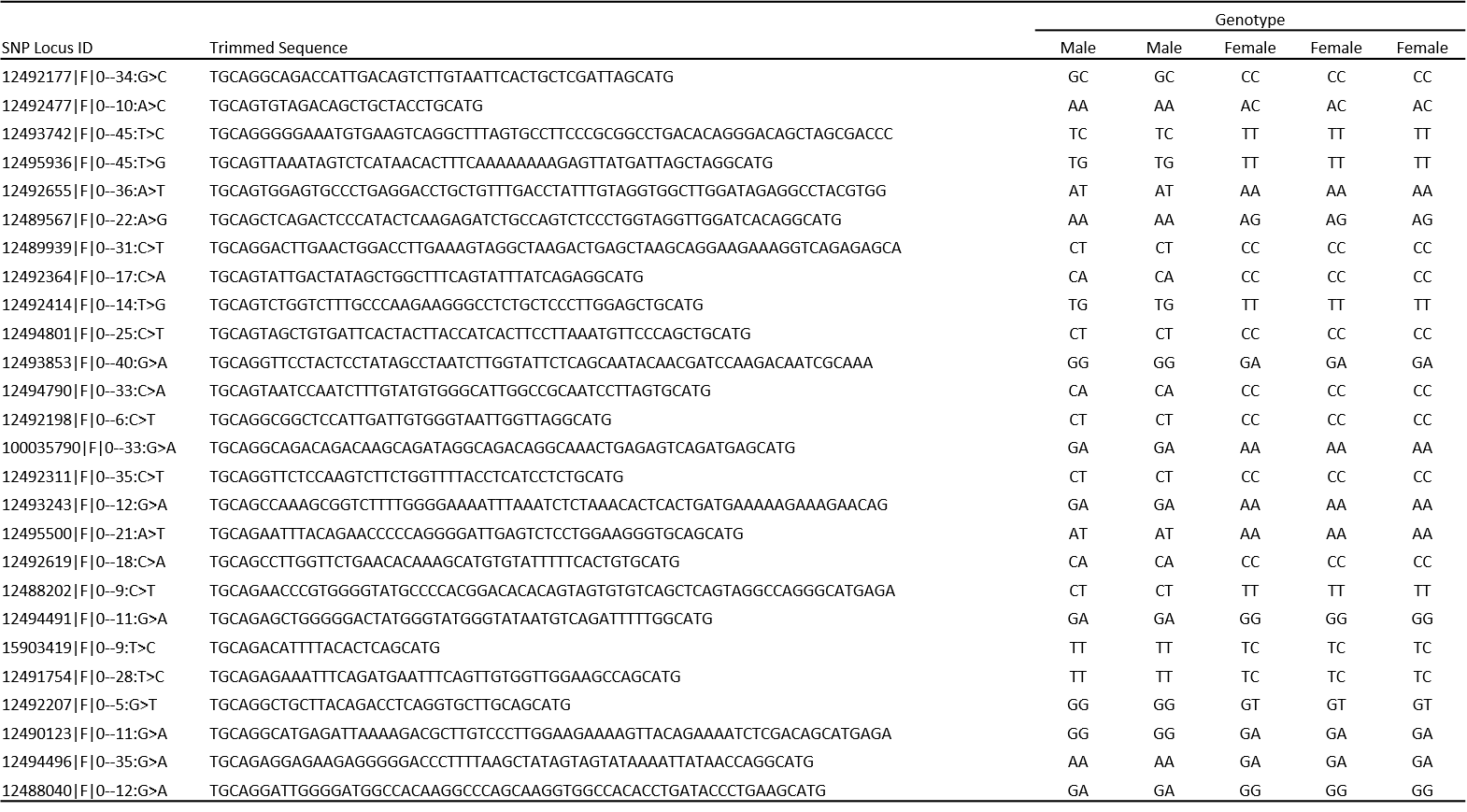


**Appendix Table 2**. BLAST results of DNA fragments from scat sequenced during koala genotyping DArTseq™ protocol. BLAST results show presence of dietary and disease information.
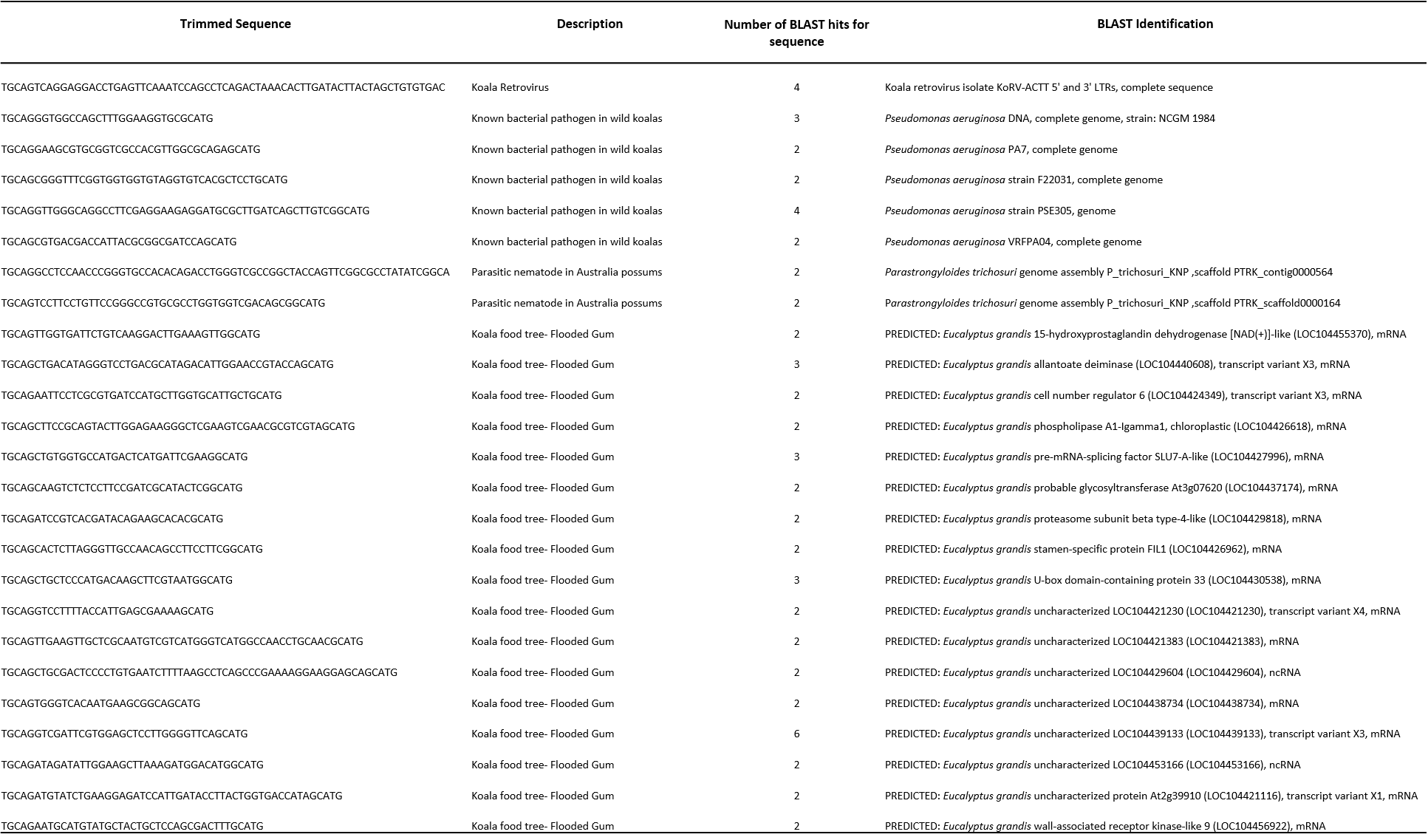


**Appendix Figure 1**. Distribution of minor allele frequencies for 1272 SNP loci, across blood DNA extracts from five individual koalas.
